# Multiplexed Single‐Cell Rheology Probing Using Surface Acoustic Waves

**DOI:** 10.1002/smsc.202300146

**Published:** 2024-02-13

**Authors:** Yi Hu, Yulin Wang, Meiru Zhang, Changkai Gao, Pu Zhao, Suyan Zhang, Zhaoguang Zan, Dachao Li, Zhenzhen Fan

**Affiliations:** ^1^ State Key Laboratory of Precision Measurement Technology and Instruments Tianjin University Tianjin 300072 China; ^2^ State Key Laboratory of Acoustics Institute of Acoustics Chinese Academy of Sciences Beijing 100190 China

**Keywords:** cell rheology measurement, power‐law dynamics, surface acoustic waves, targeted microbeads

## Abstract

Cellular rheological properties affect cell function and are reflective of cell status. It is challenging to perform multiplexed single‐cell rheology probing with high controllability, particularly for adherent cells. A surface acoustic wave (SAW)‐based method is presented for this purpose. The method integrates the potent micromanipulation ability of acoustic waves in a microfluidic chamber with the ability of cell‐anchored microbeads to concentrate the acoustic energy to deform the cell. Two strategies are developed for placing a targeted microbead at a desired position on the cell membrane. The power‐law rheological dynamics with plastic components are applied to fit the creep (during the mechanical loading) and relaxation (after force removal) responses of the cell. With more than 400 measurements of adherent cells and each with detailed dynamics, a full range of viscoelastic behaviors of cells far beyond the typical rheology of previously reported adherent cells and unexpected negative plastic compliance is observed. The developed method supports in‐depth investigations of biomechanics at the cellular and subcellular levels, with considerable potential for extension to mechanical force‐based cell function regulation.

## Graphical abstract

Multiplexed single‐cell rheology probing is achieved using surface acoustic waves (SAWs). Targeted microbeads are first located to adhered cells forming binding with cell surface proteins either on top or on the side, and then pushed by acoustic waves to deform cells for cell rheology measurement. The method allows for high‐throughput semi‐quantitative analysis of cell rheology.

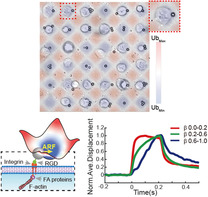

## 1 Introduction

The rheological properties of a cell play a significant role in cell function regulation and reflect the cell state during various processes such as development,^[^ ^1^ ^]^ metastasis,^[^ ^2^ ^]^ the cell cycle,^[^ ^3^ ^]^ stem cell differentiation,^[^ ^4^ ^]^ and immune activation.^[^ ^5^ ^]^ Various strategies have been developed for measuring single‐cell deformations under mechanical loads. Micropipette aspiration,^[^ ^6^ ^]^ parallel‐plate rheometry,^[^ ^7^ ^]^ optical stretching,^[^ ^8^ ^]^ microfluidics‐based methods,^[^ ^9^ ^]^ and acoustic scattering^[3b]^ have been employed to characterize the rheological behavior of cells in suspension. For most cell types in vivo, adhesion to the matrix permits survival and proper functioning. Therefore, the mechanical properties of the adherent cells detected after adhesion to substrates have far greater physiological relevance than those in suspension. Available methods for assessing adherent cell mechanics include atomic force microscopy (AFM),^[^ ^10^ ^]^ optical tweezers (OT),^[^ ^11^ ^]^ acoustic force spectroscopy (AFS),^[^ ^12^ ^]^ magnetic twisting cytometry (MTC),^[^ ^13^ ^]^ and the quartz crystal microbalance with dissipation (QCM‐D) technique.^[^ ^14^ ^]^ In addition, acoustic tweezing cytometry (ATC), which involves deflecting targeted microbubbles attached to cell membranes using traveling bulk acoustic waves, has been utilized for stem cell stiffness phenotyping during differentiation and morphogenesis.^[^ ^15^ ^]^ QCM‐D is a surface sensitive technique, whereas all other techniques use tips or microprobes with subcellular resolution. AFM has a nanometer spatial resolution, however, it is generally restricted to the cell surface and requires expensive equipment. With excellent 3D microbead positioning capability and precise force measurements, OTs are limited by heating issues and low throughput. AFS can be used to manipulate microbeads over a wide range of forces in a high‐throughput manner by forming a standing bulk wave in a chamber at resonance frequency. However, in AFS, MTC, and ATC, the attachment procedure for microprobes (microbeads, ferromagnetic beads, or microbubbles) often lacks control, leading to random cell‐probe attachment outcomes.

Because most cell types in vivo are adherent, it is desirable to develop a multiplexed single‐cell rheological characteristic probing platform suitable for adherent cells with high controllability. The most prominent differences between a cell in suspension and the cell after adhesion to the substrate are the development of a cytoskeletal protein network and the formation of focal adhesions between the cell membrane and the substrate, accompanied by changes in cell morphology, cell functions, and cell mechanics. Compared with the measurement of cells in suspension, the simultaneous multiplexed measurement of single adherent cells requires more steps, starting from the formation of adhesions and the measurement capability over a certain area, covering all the adherent cells. The integration of surface acoustic waves (SAWs) with microfluidic chambers has become an appealing approach for manipulating micron‐size objects. Two counter‐propagating surface waves generated by interdigitated transducers (IDTs) pairs deposited on a piezoelectric substrate radiate energy into a fluid in contact with the substrate and stable standing longitudinal waves are established in the fluid in the horizontal plane, trapping particles by the acoustic radiation force (ARF) generated by the second order of pressure field. The acoustic field or the particle trapping pattern can be fine‐tuned by the SAW frequency,^[^ ^16^ ^]^ frequency difference between IDTs pairs,^[^ ^17^ ^]^ and harmonic compositions in multi‐harmonic waves.^[^ ^18^ ^]^ The roof of the microfluidic chamber reflects longitudinal waves in the fluid, yielding partial standing waves perpendicular to the substrate, which can constrain the vertical motion of the particles.^[^ ^19^ ^]^ Through adjustment of the frequency, amplitude, and phase, SAW‐based methods have been used for bioparticle trapping,^[^ ^20^ ^]^ translation of cells,[^17^, ^20^] and 3D tissue assembly.^[^ ^19^, ^21^ ^]^ Although the positions of cells have been exquisitely and programmably handled in these studies, the living features of cells that distinguish cells from any other nonliving microscale objects have not been explored using SAWs.

In this study, multiplexed single‐cell rheology probing after cell adhesion was performed by deflecting integrin‐anchored microbeads using standing waves. After individual cells were patterned and adhered to the substrate, the individual targeted microbeads were brought into contact with the cell membrane, from either the top or the side, using two highly controllable and tunable strategies, yielding unprecedented selectivity of the targeted microbead‐binding position on the cell membrane. A phase lag was then applied to push the cell membrane‐attached microbeads to deform the cell. The displacement–time curves of the microbeads were fitted using power‐law dynamics. With more than 400 measurements of adherent cells, each with detailed dynamics, we observed considerable heterogeneity in the cell rheological properties, ranging from highly elastic to highly viscous. The plasticity was nonnegligible. Moreover, in many cases, the plasticity had negative values. The measurement results depended on the positions of the microbeads on the cell membrane. Owning to its high operational throughput and controllability, the developed SAW‐based method is promising for studying cellular biomechanics and for regulating cell fate mechanically.

## 2 Results and Discussion

### 2.1 Strategies for Microbead Attachment at Desired Positions

**Figure 1:**
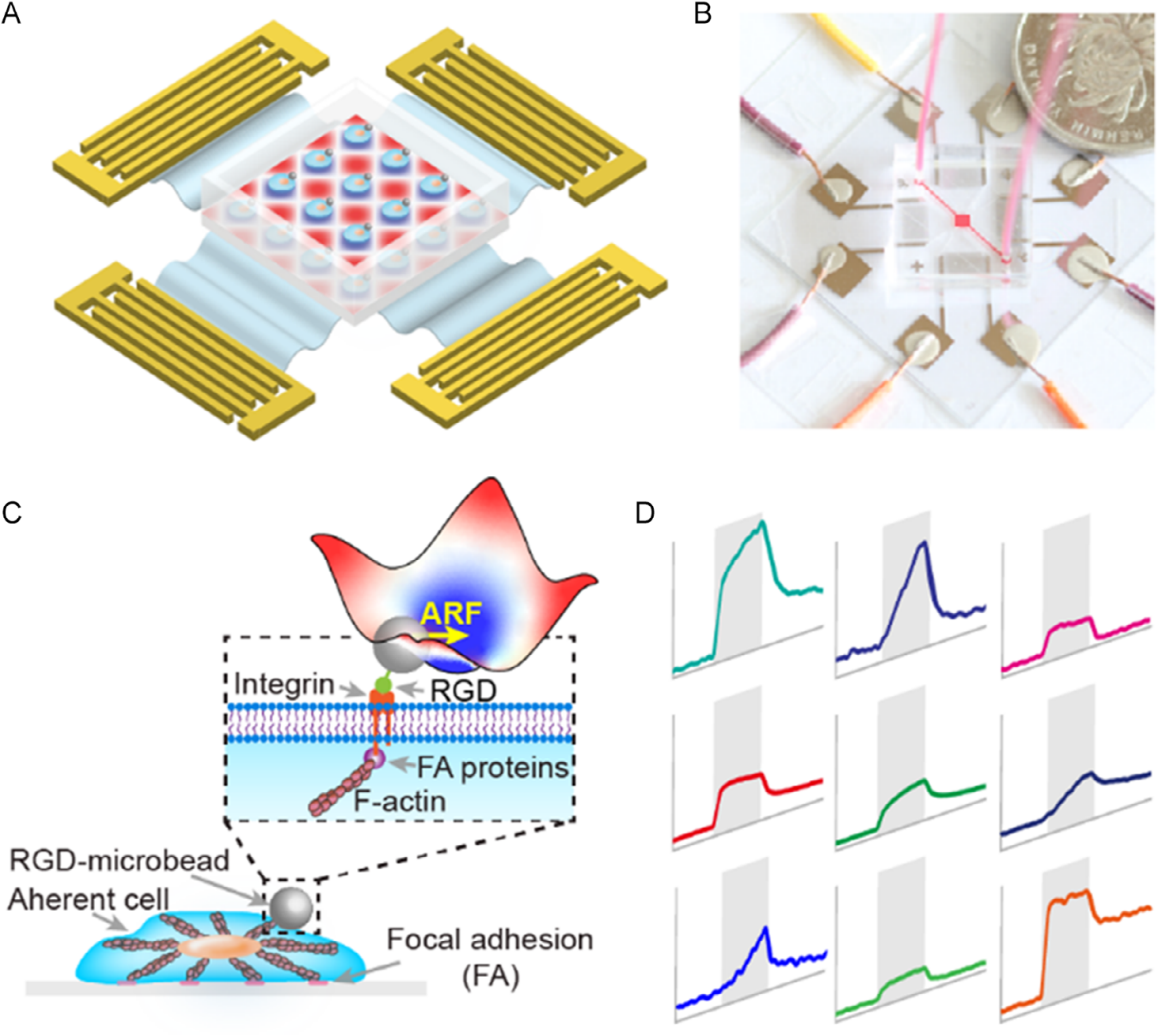
Multiplexed single‐cell rheology probing was achieved using SSAWs. A) Acoustofluidic device was designed for single‐cell rheology probing. B) Photograph of our device. C) To deform the cell, the cell membrane‐anchored microbead was pushed by ARF (mechanical loading). D) The displacement of microbeads, which reflected cell deformation, was imaged using a high‐speed camera. Multiple deformation–time curves were obtained simultaneously.

We first applied 2D standing SAWs (SSAWs) to achieve one‐cell‐one‐microbead patterning, followed by deforming cells under mechanical loading via microbeads (**Figure** **1**). The standing Rayleigh SAWs generated on the surface of the LiNbO_3_ substrate radiated energy into the fluid inside the polydimethylsiloxane (PDMS) microfluidic chamber, forming standing longitudinal waves in the fluid (Figure S1, Supporting Information). The diameter of the common mammalian cells, e.g., NIT/3T3 fibroblasts and human embryonic kidney 293 (HEK293) cells, was ≈15–20 μm in suspension and increased after adhesion and spreading. To balance the competing effects of the primary ARF trapping cells and the inter‐cells attractive secondary ARF (Bjerknes force), a wavelength of 80 μm was selected for the SSAWs, as the ratio of the wavelength to the particle diameter was suggested to be around 4.^[^ ^16^ ^]^ Arg–Gly–Asp (RGD) peptide is commonly used to facilitate cell adhesion by binding to cell surface protein integrins. RGD‐decorated polystyrene microbeads with a 5.3 μm diameter, which can anchor on the cell membrane via RGD‐integrin binding, were selected as probes (Figure 1C). Compared with microbubbles, which were used in another study for acoustic cell mechanical phenotyping,^[15a]^ uniformly sized polystyrene microbeads can significantly improve the controllability, as the inhomogeneity of the bubble size distribution and the difficulty in overcoming the buoyancy of the gas bubbles are eliminated. The ARF on a 5.3 μm diameter polystyrene bead in standing waves inside the acoustofluidic device could be higher than that on a 5.3 μm diameter microbubble in traveling bulk acoustic fields used in ATC,^[15a]^ which compensates for the weaker ability of microbeads to scatter acoustic waves compared with gas‐filled microbubbles. Moreover, the uniform size distribution of the polystyrene beads benefits the high‐throughput operations, such as the multiplexed single‐cell rheology probing performed in this study (Figure 1D). The density of polystyrene, which is slightly larger than that of water, allows 3D manipulation in a fluid because it can descend via gravity or be levitated by acoustic streaming in an acoustofluidic chamber.^[^ ^19^ ^]^

By superimposing orthogonal SSAWs with slightly different frequencies (44.8 MHz in x‐ and 44.9 MHz in y‐directions) and the same pressure amplitude, a dot‐like pressure nodes array was generated,^[^ ^17^ ^]^ allowing independent phase‐lag tuning in the *x*‐ or *y*‐direction. A disposable 1.2 mm × 1.2 mm × 100 μm PDMS square closed chamber with a 25 μm thick PDMS bottom layer was bonded to the LiNbO_3_ substrate at the middle of two pairs of IDTs (Figure 1B and S1, Supporting Information). The thin PDMS bottom layer allowed the whole PDMS chamber to be peeled off easily from the LiNbO_3_ substrate. The disposable PDMS chamber prevented cells contamination due to chamber reuse. The chamber contained 30 × 30 pressure nodes, permitting multiplexed operation.

**Figure 2:**
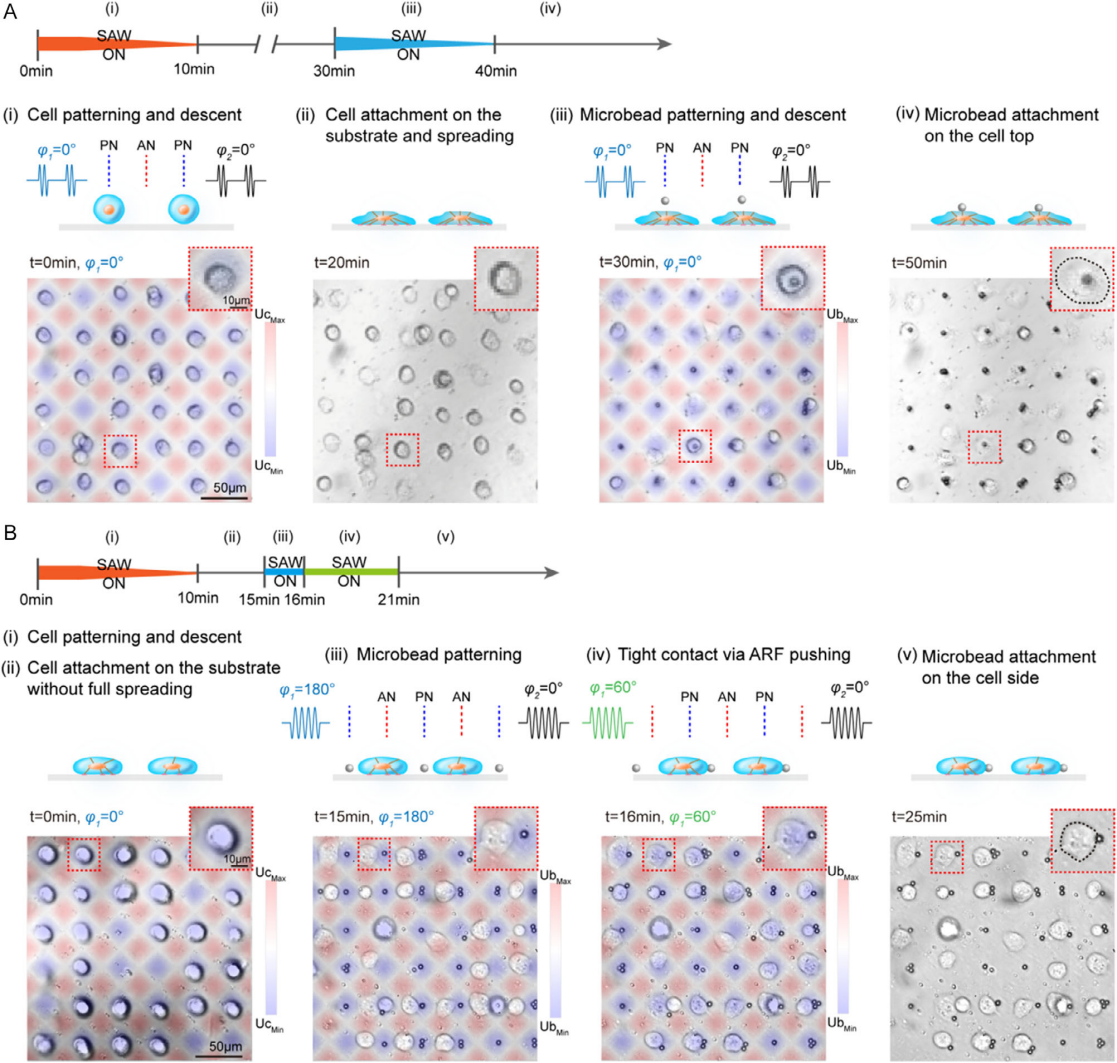
A) Apical and B) side microbead attachment approaches. Illustration of key steps and representative microscopic images of each step.

We developed two strategies for controlled microbead attachment to cells, from the top and side (**Figure** **2**). In both strategies, the cells were first patterned using SSAWs with a 0° phase lag. To protect the cells from heat damage (Figure S2, Supporting Information), pulsed signals (input voltage of 5 V, duty cycle of 20%) were employed to trap the cells. Three minutes later, after all the cells were successfully patterned, the input voltage was gradually decreased until completely turned off at 10 min to lower the cells until they were completely placed on the PDMS membrane. In the first strategy (Figure 2A), cells were allowed to adhere to the substrate for 20 min, spread and express integrin. RGD‐decorated microbeads were then patterned right above individual cells using SSAWs with a 6 V input voltage and 0° phase lag. Again, the voltage was gradually decreased until completely turned off for lowering the microbeads toward the apical cell membrane. The difference between the gravitational force and buoyant force of the microbead kept it in contact with the cell membrane until the integrin‐RGD bindings were formed.

Another strategy was to attach the microbeads to the sides of the cells (Figure 2B). After the cells were patterned and adhered to the substrate, RGD‐decorated microbeads were horizontally patterned in the middle of two cells using SSAWs with a 180° phase lag and a 4 V input voltage. The trapped microbeads were translated toward cells when the phase lag was changed to 60°. Pushing by ARF, the microbeads maintained close contact with the cell membranes, allowing the integrin‐RGD binding. Five minutes later, the SSAWs were turned off and the majority of the microbeads were linked to the cell membranes. Considering the height of cells during spreading (approximately 5–10 μm), a low input voltage (4 V) was employed to avoid lifting microbeads too high to meet cells vertically. A 120° net phase lag was applied to push the microbeads so that they were subjected to the maximum ARF (at approximately 90° phase lag) when in contact with the cell membrane.

Compared with the difference between gravity and buoyance used in the first strategy or the buoyant force used for attaching microbubbles in ATC, the ARF used in the second strategy for cell‐microbead attachment could be larger, beneficial for strong binding. However, the flattening of cells during spreading leads to a stricter requirement for the operation timing to achieve high‐efficient attachment. These two attachment strategies are complementary; thus, both apical and side cell membranes are accessible for controlled microbead attachment. Even though the final microbead attachment position on the cell membrane is inevitably affected by the cell morphology, which changes continuously during adhesion and spreading, the controllability achieved here allows the desired microbead attachment on single cells, which is difficult to achieve in random microprobe attachment procedures in MTC^[13a–c]^ and ATC.^[15a]^

Controllable and arbitrary microbead attachment to an adherent cell, the first step in cellular property probing or cell function regulation, is crucial for the entire method and determines whether this method has development potential to extend its applications and whether it can be widely adopted by related scientific communities. Microbeads can be transported to any desired position on the cell membrane, opening the door for future studies where the stimulation position is a pivotal parameter. For example, neurons with a dramatically polarized morphology may be highly sensitive to the subcellular probing locations. Moreover, recent progress in stem cell‐based embryo models has revealed a tremendous amount of positional information regarding cell sorting, protein expression, and gene transcription.^[^ ^22^ ^]^ With a 3D targeting ability, the application of the developed method to 3D embryoids, either for mechanical phenotyping or for active stimulation, is highly likely to provide fundamental knowledge about early human embryogenesis.

### 2.2 Single‐Cell Rheological Properties Probing

**Figure 3:**
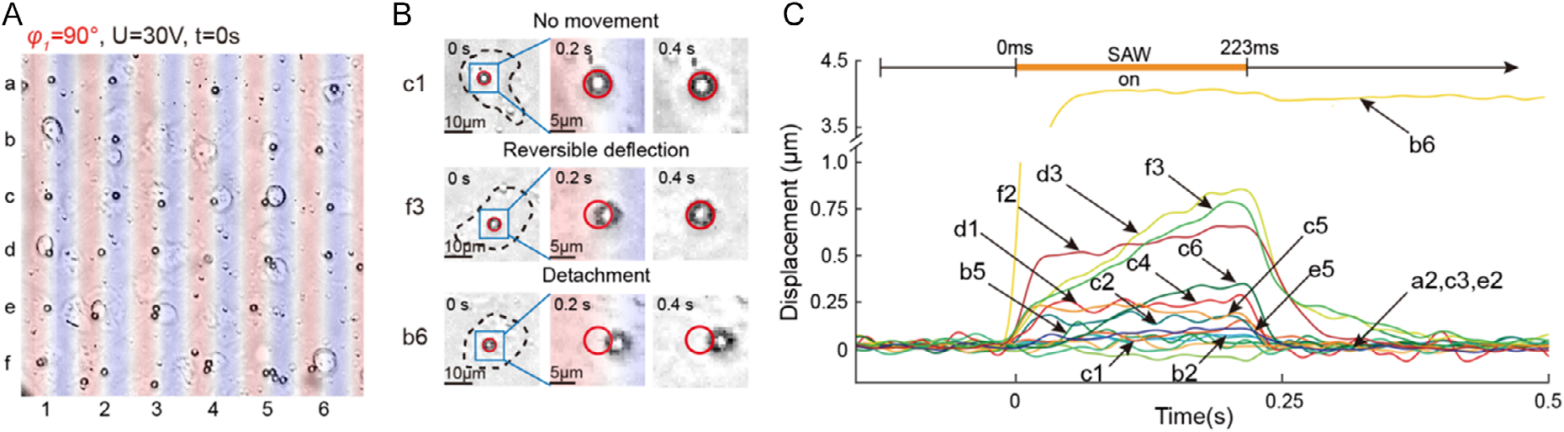
Multiplexed microbead‐mediated cell rheology probing. A) SSAWs in the x‐direction with a 90° phase shift and a 30 V input voltage were applied to deform adherent cells via microbeads. B) Selected microscopic images of microbeads at c1 (no movement), f3 (reversible deflection), and b6 (detachment) over time. C) Displacement–time courses of all 16 cell‐bound microbeads in (A).

In the field of view shown in **Figure** **3**A, 16 one cell‐one microbead pairs were successfully assembled in a 240 μm ×240 μm area. The cells were then deformed by the microbeads driven by a brief SSAW pulse (phase lag of 90°, input voltage of 30 V, and duration of 223 ms). Figure 3B shows selected microscopic images of the microbeads at c1, f3, and b6 over time, which are typical examples of no movement, reversible deflection, and detachment, respectively. The displacement–time courses of each cell‐bound microbead shown in Figure 3A are presented in Figure 3C. Eight microbeads exhibited deflection (the maximum displacement during SSAWs was more than twice of the noise level before SSAWs) during the SSAW‐on period before retracting back to their initial positions after the SSAWs were turned off, which was due to the strain recovery generated in the microbead‐RGD‐integrin‐cytoskeleton linkage. The exception was microbead b6, which was pushed toward the acoustic potential well and completely detached from the cell. Out of the total 437 measurements, 360 microbeads experienced reversible deflection, 72 exhibited no movement, and 5 were detached. When cells were fixed with 4% formaldehyde, the primary amino groups were crosslinked so that the cells became far more rigid, resisting deformation upon mechanical loading. The maximum displacement of microbeads attached on fixed cells (0.16 ± 0.06 μm, *n* = 33) was significantly less than cells under natural conditions (0.47 ± 0.40 μm, *n* = 437, *p* = 1.28e‐5 using unpaired two‐tailed student's tests).

**Figure 4:**
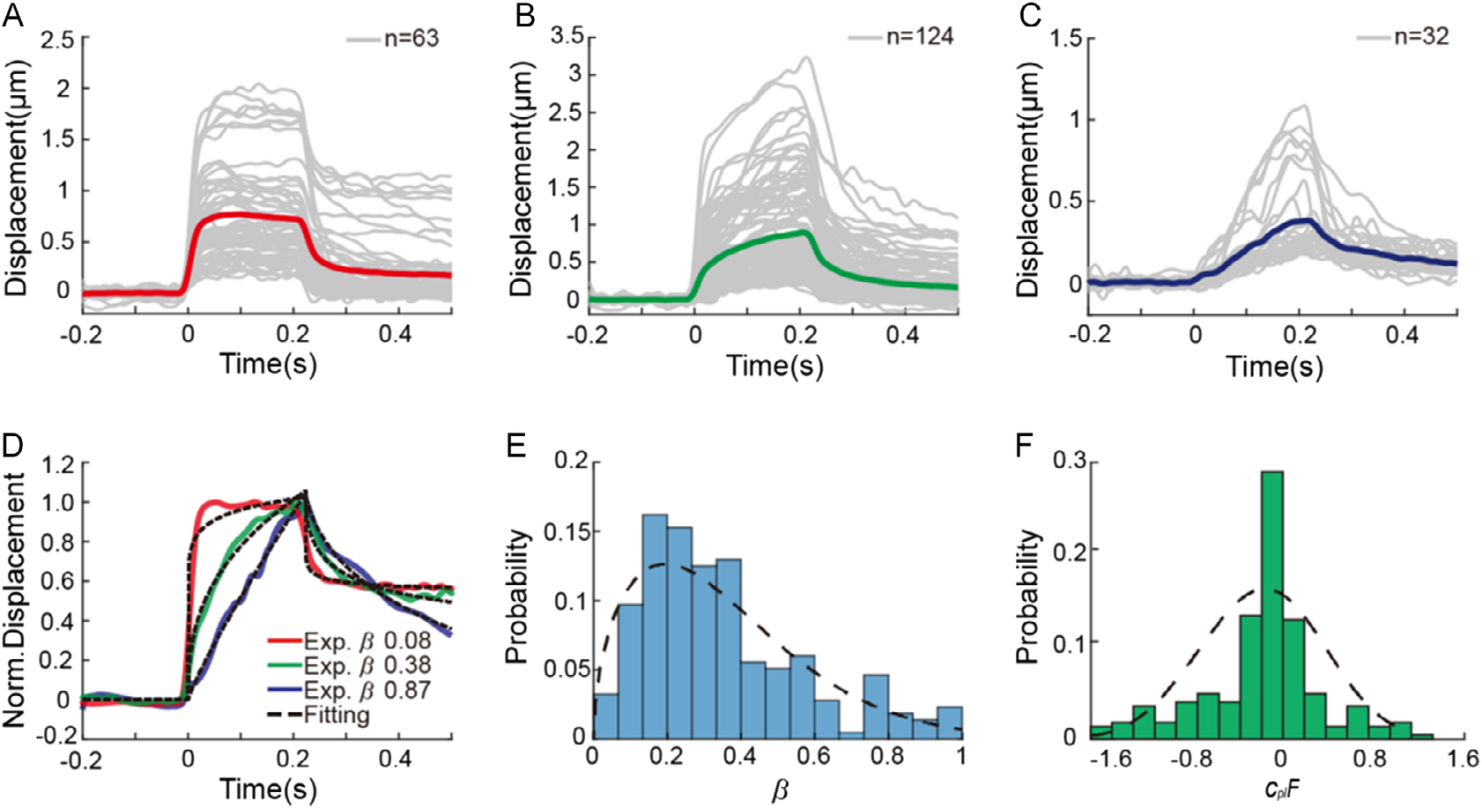
Creep and relaxation responses of single adherent cells analyzed according to the power‐law rheological dynamics with a plastic component. The 219 reversible deflection curves, whose fitting coefficients *R* ^2^ were more than 0.90, were categorized into three groups: A) with the power‐law exponent *β* between 0 and 0.2, B) between 0.2 and 0.6, and C) between 0.6 and 1. D) Representative curves obtained experimentally for each group and their fitting curves with R^2^ more than 0.98. E) The probability distributions of the power‐law exponent *β* and F) the product of the plastic compliance and the force *c*_pl_ *F* for 219 curve fittings.

In the case of reversible deflection, the translation and retraction of the cell‐bound microbeads during and after SSAW application reflected the deformation of the cell under a mechanical load (creep) and recovery after force removal (relaxation). Therefore, we used power‐law rheological dynamics to fit the microbead displacement–time curves for creep and relaxation using Equation (1).^[13b]^

(1) $$d(t)=\{\begin{array}{l} (c_{\text{ve}}+c_{\text{pl}})F{\left(t/t_{0}\right)}^{\beta },0<t\leq t_{1}\\ c_{\text{ve}}F[{\left(t/t_{0}\right)}^{\beta }-{\left((t-t_{1})/t_{0}\right)}^{\beta }]+c_{\text{pl}}F{\left(t_{1}/t_{0}\right)}^{\beta },t>t_{1} \end{array}$$

here, *d*(*t*) represents the deformation of the cell subjected to force *F*, which is applied from *t* = 0 to *t*_1_; *c*_ve_ and *c*_pl_ represent the viscoelastic and plastic cell compliances, respectively; and *t*_0_ represents the timescale, which was set to 1 because the power‐law behavior is timescale invariant.^[^ ^23^ ^]^ The power‐law exponent *β* describes the viscoelasticity of the cell, with zero indicating pure elastic deformation and unity indicating pure viscous deformation. The *β* values obtained from 219 curves, whose fitting coefficients *R* ^2^ were more than 0.90, spanned from zero to unity with the peak at ≈0.2. According to the *β* values, 219 curves were categorized into three groups, with *β* between 0 and 0.2, between 0.2 and 0.6, and between 0.6 and 1. The curves in each group exhibited distinctive features during creep responses: a sharp increase followed by stabilization, similar to that for an elastic solid (**Figure** **4**A); an exponential increase (Figure 4B), which was reported as the typical mechanical behavior of adherent cells;^[^ ^23^ ^]^ and a linear increase, similar to that for a viscous fluid (Figure 4C). In contrast to the diverse creep reactions, the relaxation dynamics were similar (Figure 4A–C). We found that to accurately fit the experimental observations during and after force application, a plastic component (*c*_pl_) was necessary in addition to the viscoelastic part (*c*_ve_). Interestingly, we observed not only positive values of the plastic compliance *c*_pl_, indicating incomplete recovery,^[13b]^ but also negative values (*F* is always positive), suggesting that the recovery was quicker and steeper than that without the plastic component (Figure 4F).

It is intriguing that measured *β* values of more than 40% of cells in this study were less than 0.2 or larger than 0.6, beyond the typical *β* values of cells reported in the literature.^[^ ^23^ ^]^ The multiplexed measurement efficiency of the developed system contributed to the unveiling of the full range of the rich viscoelastic behavior of cells. The cells probed in this study were in the early stages of adhesion, whereas the adherent cells measured in previous studies were fully settled. The wider viscoelastic spectrum captured in this study implies that adhesion begins with a highly dynamic adaptive procedure. In the future, it is feasible to perform the measurement when the cells are fully settled, as the acoustofluidic chamber is highly biocompatible, allowing cell culturing over several days. A positive value of *c*_pl_ has been used to explain the residual deformation during the relaxation, which is responsible for energy dissipation.^[13b]^ The proposed molecular mechanisms are the bond slippage or breakage in the cytoskeletal protein networks. The negative plastic composition implies that the cell will eventually exhibit a net negative deformation given sufficient relaxation time in the opposite direction to the applied force. A previous study using an optical stretcher reported a prediction of negative deformations during recoveries using a power‐law rheology model.^[^ ^24^ ^]^ The authors speculated that this corresponded to a transient higher effective stiffness, related to the soft glassy rheology model. In this study, both positive and negative values of *c*_pl_ were obtained. Whether the negative values were scatters around zero, or they reflected some transient biological processes needs further investigations. Cells are highly non‐linear living materials that stiffen or soften under mechanical stress.^[^ ^25^ ^]^ It is possible that similar to incomplete shape recovery, some unknown transient process may be another adaptive and protective strategy for cells to resist mechanical damage. It is also worth mentioning that for recovery rheology detection after force cessation, polystyrene microbeads may be able to provide more faithful measurement than the heavy microbeads, such as the magnetic microbeads used in MTC. The heavy microbeads may pose non‐negligible resistance for cells to pull them toward the original positions before force application.

**Figure 5:**
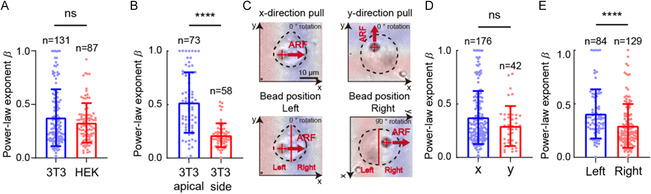
The power‐law exponent *β* was independent of the cell type but dependent on the microbeads attachment positions. A) There was no significant difference in the *β* values measured in 3T3 fibroblasts and HEK 293 cells. B) The *β* values measured from the apical microbeads were higher than those measured from the side microbeads. C) Representative microscopic images showing pushing in the *x*‐ or *y*‐direction during the experiment and images after alignment of the pushing in the *x*‐direction from left to right. D) There was no significant difference in the *β* values measured under pushing in the *x*‐ and *y*‐directions. E) The *β* values measured from the left side were higher than those measured from the right, after alignment of the pushing in the *x*‐direction from left to right. Unpaired two‐tailed student's tests were applied for statistic analysis (ns *p* > 0.05, ****p < 0.0001).

We also studied the dependence of the cell rheology measurement results on the cell type and microbead position. The rheological properties of 3T3 fibroblasts and HEK cells did not exhibit significant differences (**Figure** **5**A), possibly owing to the similar developmental stages of the intracellular cytoskeletal protein networks during adhesion. The cell rheology measurement results were significantly different between apical and side microbead attachment. The power‐law exponents *β* calculated from the apical microbeads were distributed from zero to one, with a mean value of 0.5. In comparison, the *β* values of the side microbeads were more concentrated with a much lower mean value of 0.2 (Figure 5B). Possible contributors to the *β‐*value difference included the 3D geometry of the flattened adherent cell, the uneven distribution of the actin filament cytoskeleton, and the differences in integrin expression. Moreover, we compared the cell rheological properties measured when ARF was applied in the *x*‐ and *y*‐directions (Figure 5C). The measurement results were independent of the ARF direction (Figure 5D). However, when we reassorted the data according to the relative positions of microbeads on cells (left or right) with pushing in the *x*‐direction from left to right, the *β* values of microbeads on the left were significantly higher than those of microbeads on the right (Figure 5E). This result might be attributed to the fact that the raised‐nucleus‐containing middle area of the cell impeded the rightward movement of the left microbead.

The platform we developed here extends SAW‐based solutions for biomedical applications, from cell position operations to cell rheology probing, with the potential to further extend to mechanical force‐based cell function regulation. We demonstrated activation of mechanosensitive ion channels using our platform (Figure S3, Supporting Information). Based on the established framework, further techniques can be developed to employ acoustic holograms^[^ ^21^, ^26^ ^]^ or acoustic metamaterials^[^ ^27^ ^]^ to increase the degree of freedom of operation, establish a working modality uniquely associated with microbeads (e.g., detachment‐facilitated drug and gene delivery), and explore other types of probes (e.g., microbubbles).

### 2.3 Attempt to Quantify the Total Force on Individual Microbeads and Problem Analysis

The developed method here is a semi‐quantitative method. The power‐law exponent *β* can be obtained from the displacement–time curve fitting. However, since *F* is unknown, only the products of the viscoelastic compliance/the plastic compliance and the force *c*_ve_ *F/c*_pl_ *F* can be calculated. Actually, we have tried very hard to provide precise total force calculation on individual microbeads aiming to develop this method fully quantitative. However, after careful examination of all factors, we believe it is, currently, impossible to provide a correct determination of the total force on microbeads attached on cells.

We first recorded the motion of 4.6 μm diameter‐naked‐polystyrene beads at *z* = 5 μm driven by a phase lag. We tried to fit the displacement–time courses with the analytical solution with the drag force induced by the velocity of the acoustic streaming neglected with the goal to calculate the acoustic pressure at the pressure nodes. However, the drag force induced by the velocity of acoustic streaming at *z* = 5 μm is highly likely to be more than 1% of the ARF, not negligible. Therefore, no analytical solution is available to fit the displacement–time course of microbeads at *z* = 5 μm.

The second problem is about the simulation. The layout of IDTs on 128°Y LiNbO_3_ rotated by ±45° has the advantages of the same coupling coefficients and SAW velocities in *x*‐ and *y*‐directions. Therefore, this setting has been widely used in acoustomicrofluidic manipulation. However, a non‐neglectable beam steering effect is associated with this layout due to the anisotropic orientation‐dependent SAW propagation properties. For one directional SSAW with this setting, the power flow angle was reported to be 4.48°.^[^ ^28^ ^]^ Since the standing waves are established in *x*–*y* and *x*–*z* directions inside the chamber, a 3D simulation, which contains 900 pressure nodes, is sufficient to calculate standing waves in two directions and to include the beam streaming effects. This calculation would consume enormous computational power, if not completely impossible. A previous study suggested a velocity boundary condition for the fluidic domain to replace the LiNbO_3_ substrate to simplify the calculation.^[^ ^29^ ^]^ In our device, a 25 μm thick PDMS layer was employed on top of the LiNbO_3_ substrate and beneath the fluidic domain. Whether this velocity boundary condition is still applicable in our setting needs further validation.

Finally, the presence of single cells strongly affects the local acoustic field and acoustic streaming field, making the quantification of the total force on microbeads completely impossible. The acoustic properties of cells are different from the surrounding liquid. Moreover, the acoustic properties inside cells are neither homogeneous nor static. The size of a cell is close to that of the acoustic potential well established. Thus, the acoustic field and acoustic streaming field are altered dramatically near cells. Another important player that complicates the total force determination on microbeads is the secondary ARF between a microbead and a cell, which was estimated to be one order of magnitude smaller than the primary ARF using equations for two spheres with different properties in contact in a standing wave.^[^ ^30^ ^]^ When a microbead is on top of a cell, the secondary ARF is almost perpendicular to the *x*‐directional pulling ARF. In contrast, when a microbead is attached on the side of a cell, there is a large projection of the secondary ARF in *x*‐direction, contributing greatly to the total force on microbeads in *x*‐direction.

Taken altogether, it is not possible to provide accurate total force calculation on individual microbeads in current design. If SSAWs with a longer wavelength are applied, the drag force induced by the acoustic streaming and the secondary ARF between a microbead and a cell would decrease. However, the primary ARF would decrease as well. If traveling bulk waves with a low frequency, i.e., 1 MHz, are used instead of SSAWs to drive the displacement of microbeads, a much more homogeneous time‐averaged acoustic pressure field would be established inside the chamber, substantially simplifying the calculation of the total force on microbeads. However, without the gradient of acoustic potential in standing waves, the primary ARF on polystyrene microbeads in traveling waves would become much smaller. An approach that might permit correct force determination is to employ microbubbles instead of microbeads in traveling bulk acoustic waves with a low frequency, i.e., 1 Mhz, in which the primary ARF of microbubbles is about nine orders of magnitude larger than that of microbeads with the same diameter. And the acoustic streaming would be significantly suppressed. However, the secondary ARF between a microbubble and a cell would be much larger than that between a microbead and a cell. If the secondary ARF is significantly smaller than the primary ARF on a microbubble, quantification of the total force on a microbubble is possible. Further investigation is needed to validate this condition. Moreover, in order to use microbubbles, uniform‐sized microbubbles need to be produced first and the buoyancy of microbubbles need to be conquered in assembling one microbubble‐one cell complexes.

## 3 Conclusions

In this study, we achieved single‐cell rheological probing of adherent cells facilitated by targeted microbeads using SSAWs with high throughput and high controllability. These technique features are advantageous compared with alternative microprobe‐mediated cell rheological measurement methods. Through contactless, dexterous, and sequential 3D manipulation, the anchor positions of the microbeads on the cell membrane can be arbitrarily controlled. The multiplexed SAW‐based platform that we developed is particularly suitable for discovering overall trends in a cell population with individual differences. Our findings offer new insights into the rheology of adherent cells and suggest a highly dynamic and adaptive early stage of cell adhesion. We envision that the implementation of our multiplexed SAW‐based single‐cell operating platform will significantly advance our understanding of mechano‐sensing, ‐responsiveness and ‐transduction at the subcellular, cellular, and organoid levels.

## 4 Experimental Section

4.1

4.1.1

##### Device Fabrication

Two pairs of IDTs, each consisting of a 200 nm thick Al conductive layer were deposited orthogonally on an 128° Y‐cut lithium niobate (LiNbO_3_) substrate at a 45° angle in the X‐direction. Each set of IDTs consisted of 40 pairs of electrodes with a width and spacing gap of 20 μm. A disposable closed microfluidic chamber, which was composed of a top square PDMS channel having a width of 1.2 mm and height of 100 μm and a 25 μm thick bottom layer, was bonded onto the LiNbO3 substrate. The PDMS chamber had 160 μm thick side walls to maximize the acoustic energy transmission into the fluidic domain inside the PDMS chamber. To promote cell adhesion on the PDMS bottom, the chamber was coated with fibronectin (20μg/mL for NIH/3T3 fibroblasts, 100 μg mL^−1^ for HEK cells; F0895, Sigma) overnight before the experiment. The reflection S11 of the IDTs was measured using a network analyzer (P9370A, Keysight).

##### Experimental Setup

The fabricated acoustofluidic device was mounted on the stage of a confocal fluorescence microscope (IX83‐FV3000, Olympus). The microbead solutions or cell solutions were manually injected into the PDMS chamber. To generate SAWs, the IDTs were driven by two‐channel arbitrary function generators (33622 A, Keysight). Power amplifiers (25A250A, Amplifier Research) were used when high input voltages (>10 V) were required. Microbead motions were recorded using a high‐speed camera (SA‐X2, Photron) at a frame rate of 1 k fps and then analyzed using a customized MATLAB code.

##### Microbead Preparation

Streptavidin polystyrene beads with 5.3 μm diameters (ZSH‐SVP‐50‐5, Spherotech) (0.5% w/v) were first mixed with biotinylated Arg‐Gly‐Asp (RGD) peptides (PCI‐3697‐PI, Peptides) (1 mg mL^−1^) for 20 min at room temperature at a volume ratio of 20:1. Before the on‐chip experiments, targeted microbeads were diluted in Dulbecco's modified Eagle's medium (DMEM) to a concentration of 1.5 × 10^7^ mL^−1^.

##### Cell Culture and MscL‐G22S Expression in HEK Cells

NIH/3T3 fibroblasts (CRL‐1658, ATCC) were cultured in a growth medium consisting of high‐glucose DMEM supplemented with 10% bovine serum (Gibco) at 37 °C and with 5% CO_2_. HEK‐293 T cells were cultured in DMEM supplemented with 10% bovine serum at 37 °C and with 5% CO_2_. For gene transfection, HEK‐293 T cells were harvested and cultured on a 60 mm Petri dish coated with poly‐L‐lysine. ≈6 h later, a mixture of 2 μg MscL‐G22S plasmid, encoding the mechanosensitive channel of large conductance, and 1.5 μg of GCaMP8f plasmid, encoding the green fluorescence calcium reporter, 8 μL of GeneJet VerII transfection reagent (Sigma) and 1 mL of DMEM was added into the Petri dish. The culture medium was changed after 16 h. The cells were ready for use after being cultured in the standard medium for another 24 h. Before the on‐chip experiment, NIH/3T3 fibroblasts or HEK cells were harvested and resuspended in fresh DMEM to an approximate concentration of 2.5 × 10^6^ mL^−1^.

##### Statistical Analysis

The statistical significance was determined using GraphPad Prism v9.0.2 (GraphPad Software Inc). Data were described by the mean and standard error of the mean (SEM). Unpaired two‐tailed student's tests were applied for comparisons between two groups. ns, statistically insignificant and *P* > 0.05. All data were considered statistically significantly different when *p* < 0.05. *, *p* < 0.05. **, *p* < 0.01. ***, *p* < 0.001. ****, *p* < 0.0001.

## Conflict of Interest

The authors declare no conflict of interest.

## Supporting information

Supplementary Material

## Data Availability

The data that support the findings of this study are available from the corresponding author upon reasonable request.
