# Supplementary material for: Multiplexed Single‐Cell Rheology Probing Using Surface Acoustic Waves
Source: Small Sci. 2024 Feb 13;4(4):2300146. doi: 10.1002/smsc.202300146 (PMC11935143; doi:10.1002/smsc.202300146)
Supplement: Supplementary file 1 — Supplementary Material [file SMSC-4-2300146-s001.pdf]

Supporting Information

Multiplexed single-cell rheology probing using surface acoustic waves

*Yi Hu, Yulin Wang, Meiru Zhang, Changkai Gao, Pu Zhao, Suyan Zhang, Zhaoguang Zan, Dachao Li, and Zhenzhen Fan**

Y. Hu, Y. Wang, M. Zhang, C. Gao, P. Zhao, S. Zhang, Z. Zan, D. Li and Z. Fan
State Key Laboratory of Precision Measurement Technology and Instruments, Tianjin University, Tianjin 300072, China.
E-mail: zhenzhen.fan@tju.edu.cn

Z. Fan
State Key Laboratory of Acoustics, Institute of Acoustics, Chinese Academy of Sciences, Beijing 100190, China.

This file includes:

Figs. S1 to S3

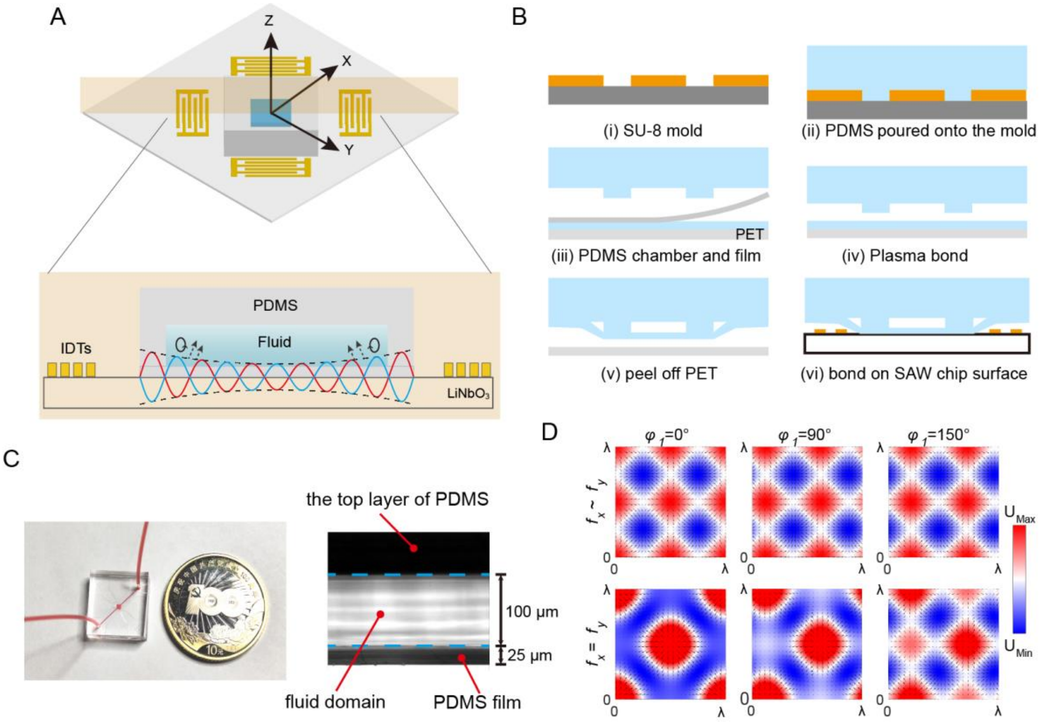

Figure S1. Working principles and designs in this study. (A) Schematic of our acoustofluidic device. SSAWs are established on the surface of $LiNbO_3$ substrate and radiate energy into the fluid domain. (B) Steps for PDMS chamber fabrication and conjugation on the SAW chip. (C) Photograph of the PDMS chamber closed with the bottom layer filled with red ink and a microscopic image of its cross-section. (D) By superimposing orthogonal SSAWs with slightly different frequencies (44.8MHz in x- and 44.9MHz in y-directions) and the same pressure amplitude, a dot-like pressure nodes array can be generated, allowing independent phase-lag tuning in x- or y-direction without pattern changes.

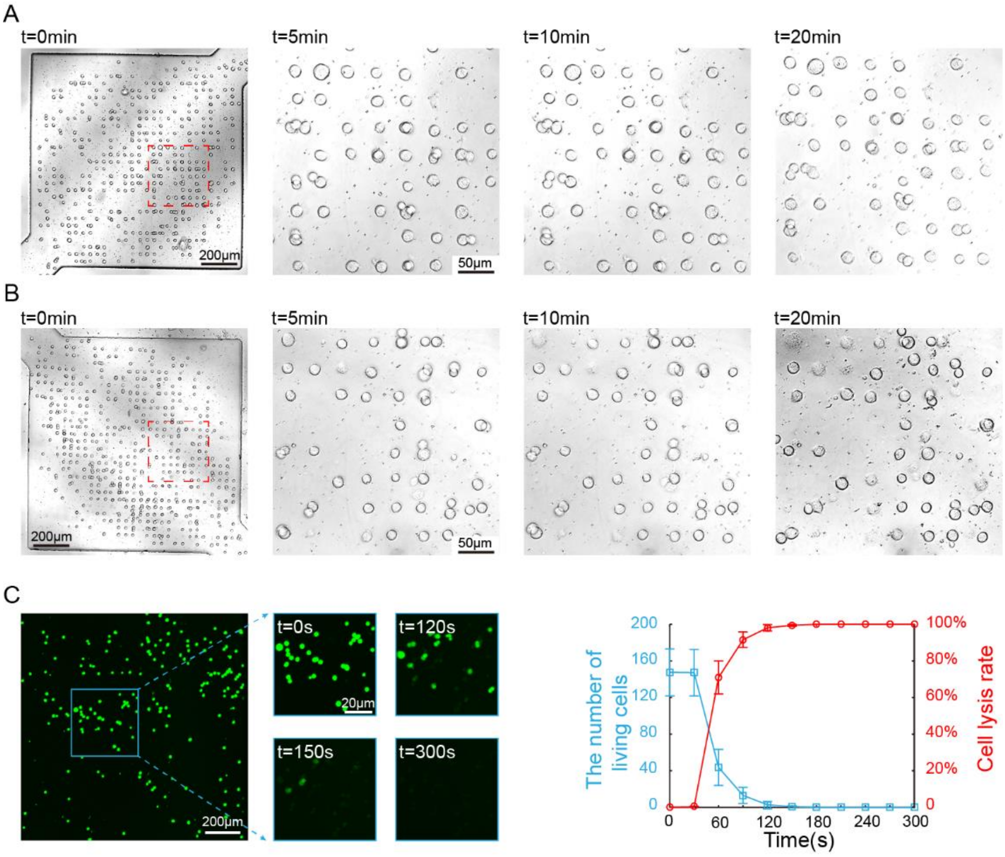

Figure S2. Cell viability with different SSAWs conditions. Bright-field images of 3T3 fibroblasts over 20min, with 1min SSAWs (input voltage of 7V, duty cycle of 10%) (A) and 2min SSAWs (input voltage of 5V, duty cycle of 20%) (B) from t=0s. (C) The fluorescence images of 3T3 fibroblasts preloaded by Calcein AM with continuous SSAWs with a 20V input voltage from t=0s. The curves of the living cell number and the cell lysis rate over time.

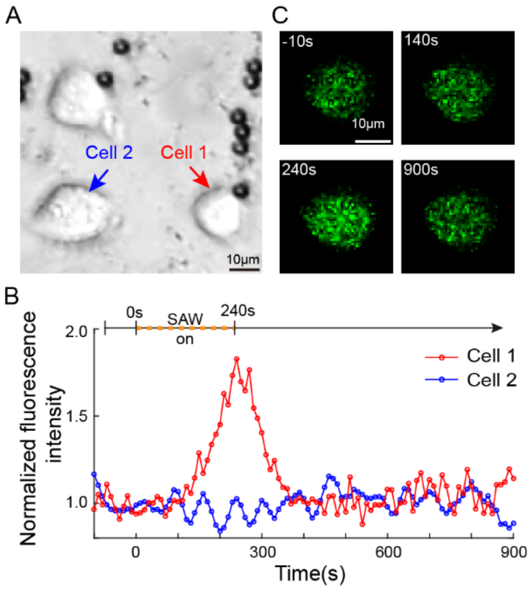

Figure S3. Mechanosensitive ion channel activation. (A) The bright field image of cell 1 and cell 2. Cells were co-transfected with the mechanosensitive channel of large conductance, MscL-G22S, and green fluorescence calcium reporter gene, GCaMP6f. Cell 1 had one microbead attached and cell 2 had no bead. (B) Normalized fluorescence intensity changes over time in both cells. Pulsed SSAWs were applied for 4 minutes (phase lag 180°, input voltage 20V, pulse repetition frequency 0.1Hz, duty cycle 2.3%). (C) Selected fluorescence images of cell 1 at different time.
